# In Vitro Stimulation with Live SARS-CoV-2 Suggests Th17 Dominance In Virus-Specific CD4+ T Cell Response after COVID-19

**DOI:** 10.3390/vaccines10091544

**Published:** 2022-09-16

**Authors:** Igor Kudryavtsev, Victoria Matyushenko, Ekaterina Stepanova, Kirill Vasilyev, Larisa Rudenko, Irina Isakova-Sivak

**Affiliations:** Institute of Experimental Medicine, 197022 Saint Petersburg, Russia

**Keywords:** SARS-CoV-2, COVID-19 convalescents, T cell memory, SARS-CoV-2-specific Th sub-sets, memory Th17

## Abstract

The SARS-CoV-2 and influenza viruses are the main causes of human respiratory tract infections with similar disease manifestation but distinct mechanisms of immunopathology and host response to the infection. In this study, we investigated the SARS-CoV-2-specific CD4+ T cell phenotype in comparison with H1N1 influenza-specific CD4+ T cells. We determined the levels of SARS-CoV-2- and H1N1-specific CD4+ T cell responses in subjects recovered from COVID-19 one to 15 months ago by stimulating PBMCs with live SARS-CoV-2 or H1N1 influenza viruses. We investigated phenotypes and frequencies of main CD4+ T cell subsets specific for SARS-CoV-2 using an activation induced cell marker assay and multicolor flow cytometry, and compared the magnitude of SARS-CoV-2- and H1N1-specific CD4+ T cells. SARS-CoV-2-specific CD4+ T cells were detected 1–15 months post infection and the frequency of SARS-CoV-2-specific central memory CD4+ T cells was increased with the time post-symptom onset. Next, SARS-CoV-2-specific CD4+ T cells predominantly expressed the Th17 phenotype, but the level of Th17 cells in this group was lower than in H1N1-specific CD4+ T cells. Finally, we found that the lower level of total Th17 subset within total SARS-CoV-2-specific CD4+ T cells was linked with the low level of CCR4+CXCR3– ‘classical’ Th17 cells if compared with H1N1-specific Th17 cells. Taken together, our data suggest the involvement of Th17 cells and their separate subsets in the pathogenesis of SARS-CoV-2- and influenza-induced pneumonia; and a better understanding of Th17 mediated antiviral immune responses may lead to the development of new therapeutic strategies.

## 1. Introduction

Influenza viruses are one of the main viral respiratory pathogens that, before the new coronavirus disease pandemic emerged in 2019 (COVID-19), caused up to 645,000 annual influenza-associated illness deaths [1]. The COVID-19 pandemic continues to cause a severe socio-economic crisis around the world, with over 560 million cases and over 6.3 million fatalities registered to date [2]. These two human respiratory pathogens generate distinct profiles of antigen-specific CD4+ and CD8+ T-cell responses upon infection [3,4], which potentially can contribute to the different levels of protection against re-infection with antigenically evolved viruses that escape from antibody immunity. It is very important to assess the levels of virus-specific CD4+ T cells responses because different Th subsets play a main part in the synergy between innate and adaptive immunity, leading to more effective and successful infection control [5]. It was shown that accelerated pathogen recognition and the early appearance of SARS-CoV-2-specific CD4+ T cells in circulation had the strongest association with the severity of COVID-19, since effective and early induction of these T cells in acute COVID-19 has resulted in a milder form of the disease with accelerated viral clearance, whereas the lack of SARS-CoV-2-specific Th cells was closely linked with severe COVID-19 (reviewed in [6]). During the acute phase of COVID-19, SARS-CoV-2-specific CD4+ T cells appeared relatively early and increased over time [7,8]. Analysis of PBMCs from COVID-19 convalescents found T cells that were specific to various SARS-CoV-2 antigens, such as Spike (S), membrane (M) and nucleoprotein (N) [9]. These circulating virus-specific CD4+ T cells were identified in 100% of patients recovered from COVID-19 [10]. Furthermore, spike-specific T cells dominated in total SARS-CoV-2-specific CD4+ T cell responses (up to 27% of the total CD4+ response), while the M and N proteins each accounted for 21% and 11% of the total CD4+ response, respectively. Other minor SARS-CoV-2 proteins, such as NSP3, NSP4, and ORF8, account on average for about 5% of the total CD4+ T cell response [11]. However, for both influenza virus and SARS-CoV-2, the use of peptide libraries encompassing certain viral proteins or predicted MHC I or MHC II restricted epitopes to stimulate PBMCs in vitro can lead to underestimation of virus-specific CD4+ and CD8+ T cell levels due to the detection of a limited T cell repertoire compared to using an unbiased peptide library [12]. In this regard, the use of live replicating viruses for in vitro stimulation of PBMCs might be particularly advantageous over stimulation with overlapping peptide pools or epitope-specific peptides because presentation of a unique pool of viral epitopes from all viral structural and non-structural proteins will be possible for each individual. Therefore, the repertoire of detected SARS-CoV-2- and influenza-specific T cells can be significantly increased. Earlier we demonstrated that the stimulation of PBMCs of COVID-19 patients with live SARS-CoV-2 and H1N1 influenza virus revealed the persistence of virus-specific IFNγ-secreting memory CD4+ T cells in circulation [13]; however, little is known about the composition of T-helper cell subsets specific to these respiratory viruses.

## 2. Materials and Methods

### 2.1. Study Participants

Nineteen COVID-19 convalescents aged 23 to 74 years participated in the current study, with disease onset ranging between March 2020 and May 2021 and time post symptoms onset (PSO) of between 1 and 15 months (Supplementary Table S1). Among them, six individuals recovered from moderate disease, accompanied by pneumonia with lung infiltrate between 25 and 50% on computer tomography. All patients were from St. Petersburg, Russia. This study was approved by the local ethical committee of the Institute of Experimental Medicine (protocol #No2/20 dated 7 April 2020), and all participants signed an informed consent form. Whole blood was collected in heparin-containing vacutainer tubes (BD, USA) for PBMC isolation and in VACUETTE™ Z Serum Sep Clot Activator Tubes (Greiner Bio-One, Frickenhausen, Germany) for serum isolation. The RBD-specific serum IgG and SARS-CoV-2 neutralizing antibodies were measured in all subjects at the time of sample collection using previously described procedures [13]. In general, the endpoint IgG antibody titers were long-lived regardless of disease severity, whereas neutralizing antibody levels decreased significantly over time in mild cases but were more persistent in subjects who recovered from COVID-19 of moderate severity (Supplementary Table S1).

### 2.2. Viruses

The SARS-CoV-2 isolate hCoV-19/St_Petersburg-3524S/2020 (GISAID EPI_ISL_415710) was obtained from Smorodintsev Research Institute of Influenza (Saint Petersburg, Russia). Virus was grown on Vero CCL81 cells using DMEM supplemented with 10 mM HEPES, 1× antibiotic-antimycotic and 2% FBS (DMEM/2%FBS). A wild-type human influenza virus A/South Africa/3626/2013 (H1N1) was purchased from NIBSC (National Institute for Biological Standards and Control, London, UK). The virus was grown in 10-day old embryonated chicken eggs. To remove contaminant proteins from the viral stocks, both SARS-CoV-2 and influenza virus were purified by ultracentrifugation on a 30%/60% sucrose gradient as described elsewhere [13]. The final precipitates containing live SARS-CoV-2 or H1N1 influenza virus were resuspended in PBS and stored in single-use aliquots at −70 °C. Infectious titers were determined either by 50% Tissue Culture Infection Dose (TCID_50_) assay for SARS-CoV-2 [14] or by end-point titration in eggs (for influenza virus), and were calculated by the Reed and Muench method and expressed in log_10_TCID_50_/mL or log_10_EID_50_/mL, respectively [15].

### 2.3. PBMC Isolation and Stimulation with SARS-CoV-2 and Influenza Viruses

Peripheral blood mononuclear cells (PBMCs) were isolated using Lymphocytes Separation Media by standard procedures (Capricorn, Ebsdorfergrund, Germany). PBMCs were resuspended in RPMI 1640 media supplemented with 10% FBS, 5 mM HEPES, 1× antibiotic-antimycotic, and 50 µM β-mercaptoethanol (CR-10 medium), and 1 × 10^6^ cells were added to each well of a U-bottom 96-well plate (Sarstedt, Nümbrecht, Germany) for further stimulation with SARS-CoV-2 and influenza H1N1 viruses. For stimulation of human PBMCs with live influenza virus, MOIs 1 to 3 are commonly used [16,17]; here, we stimulated PBMCs with H1N1 influenza virus at a dose MOI = 1. The virus was diluted in CR-0 serum-free medium and incubated with cells for 30 min at 37 °C, 5% CO_2_ for virus adsorption. Then, the CR-30 media was added to a final FBS concentration 10%. SARS-CoV-2 was used at a dose 0.001 MOI (multiplicity of infection), which was previously identified as the optimal dose for activating virus-specific T cells in the PBMC population [13]. For each PBMC sample, negative (CR-10 only) and positive (PMA/ionomycin mixture) controls were included. After 18-h incubation with both viruses at 37 °C and 5% CO_2_, a staining procedure was performed as described below. All incubation and staining procedures were performed in a biosafety level 3 laboratory by qualified personnel.

### 2.4. Activation Induced Cell Marker Assay and Flow Cytometry

Assays were conducted as previously described [18,19,20]. We evaluated SARS-CoV-2-specific T cell responses by activation induced cell marker assay and multicolor flow cytometry. The cells were stained with the following surface antibody cocktail at 4 °C for 20 min in the dark: CD69-Alexa Fluor^®^ 488 (clone FN50, BioLegend, San Diego, CA, USA), CD137-PE (4-1BB, clone 4B4-1, BioLegend, USA), CXCR5-PerCP/Cy5.5 (CD185, clone J252D4, BioLegend, USA), CCR6-PE/Cy7 (CD196, clone G034E3, BioLegend, USA), CXCR3-APC (CD183, clone G025H7, BioLegend, USA), CD3-APC-Alexa Fluor 750 (clone UCHT1, Beckman Coulter, Indianapolis, IN, USA), CD4-Pacific Blue (clone 13B8.2, Beckman Coulter, USA), CCR4-Brilliant Violet 510 (CD194, clone L291H4, BioLegend, USA), CD45RA- Brilliant Violet 650™ (clone HI100, BioLegend, USA) and CD62L-Brilliant Violet 785™ (clone DREG-56, BioLegend, USA), and ViaKrome 808 Fixable Viability Dye (Beckman Coulter, USA) for detection live/dead cells. Staining protocols were performed in accordance with the manufacturer’s recommendations. The samples were then washed twice with 200 μL of a wash buffer. Finally, the cells were fixed in 100 µL of Cyto-last buffer (Biolegend, San Diego, CA, USA) and stored in a dark cool place prior to the flow cytometric analysis. At least 500,000 events were measured using a CytoFlex LX flow cytometer (Beckman Coulter, Indianapolis, IN, USA). Single-stained samples were used for detector sensitivity calibration, as well as for compensation matrix calculation using autocompensation tools of the flow cytometer; and the resulting compensation matrix was validated with the FMO controls. The data were analyzed using Kaluza software (Beckman Coulter, Indianapolis, IN, USA); CD137+CD69+ cells were considered SARS-CoV-2-specific cells among CD4+ T cells. CD137+CD69+ gates were drawn relative to the unstimulated condition for each donor. The gating strategy for the main CD4+ T cell subsets was described in detail previously [21], and the main steps of analyses are shown on Figures S1–S3, along with representative gates.

### 2.5. Statistical Analysis

Data were analyzed using Statistica 7.0 (StatSoft, Tulsa, OK, USA) and GraphPad Prism 8 (GraphPad software Inc., San Diego, CA, USA) software. Pearson’s chi-squared test was used to assess the normality distribution of the data. The data were plotted as median and interquartile range: Me (25; 75). The differences between groups were analyzed using a nonparametric Mann–Whitney U-test. The relationship between month PSO and relative number of different CD4+ T cell subsets was evaluated by the Spearman rank correlation test, and *p* < 0.05 was considered significant. To perform t-SNE analysis, the cell fluorescence data obtained from H1N1 and SARS-CoV-2-stimulated samples were combined into two groups corresponding to the used stimulation protocol, and a random sample (*n* = 800) was selected from each group. A t-distributed Stochastic Neighbor Embedding analysis (t-SNE) was performed using R version 4.0.0. The perplexity parameter was set to 40. The iteration number was 1500.

## 3. Results

This study involved a cohort of patients who had recovered from COVID-19 because we had previously demonstrated that these patients had high levels of CD4+ memory T cells specific to SARS-CoV-2- and H1N1 influenza, whereas COVID-19 naïve subjects had influenza-specific but not SARS-CoV-2-specific responses [13]. To investigate the SARS-CoV-2-specific CD4+ T cell response, we stimulated freshly isolated PBMCs with live SARS-CoV-2 or H1N1 influenza viruses, and CD137+CD69+ cells were considered virus-specific cells among CD4+ T cells. Circulating SARS-CoV-2 memory CD4+ T cell responses were quite robust (CD137+CD69+ were clearly detected in all 19 samples), but the related number of SARS-CoV-2-specific cells within total CD45RA– memory Th cell subset was lower than the related number of H1N1-specific Th cells (0.06% (0.04; 0.11) vs. 0.15% (0.11; 0.32), *p* < 0.001).

**Figure 1:**
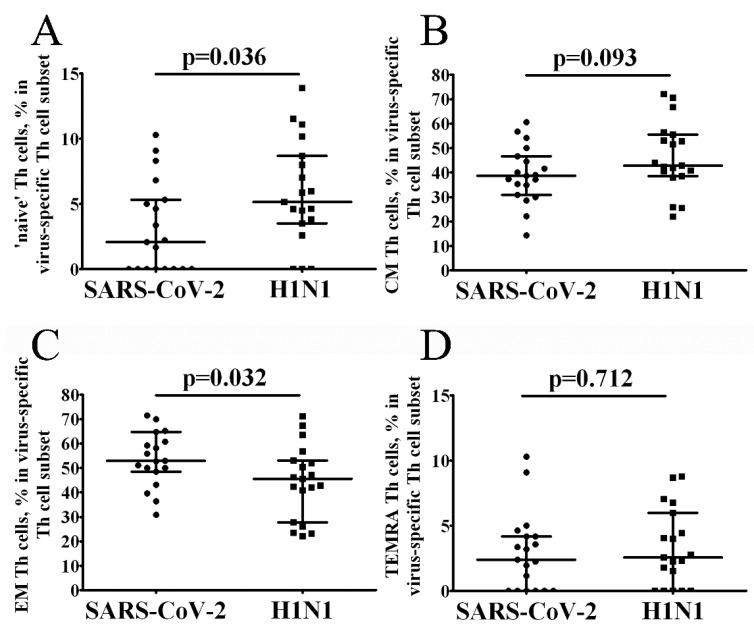
Distribution of ‘naïve’, central memory, effector memory and TEMRA cells among total SARS-CoV-2- and H1N1-specific CD4+ T cells. Scatter plots A-D showing the percentages of ‘naïve’ (**A**), central memory (**B**), effector memory (**C**) and TEMRA (**D**) cells among the total virus-specific Th cell population, respectively. Black circles—SARS-CoV-2-specific CD4+ T cells (*n* = 19); black squares—H1N1-specific CD4+ T cells. Each dot represents individual subjects, and horizontal bars represent the group medians and quartile ranges (Med (Q25; Q75). A statistical analysis was performed with the Mann-Whitney U test.

Next, we examined the differentiation status of SARS-CoV-2- and H1N1-specific Th cells based on CD45RA and CD62L expression on their cell surface. We distinguished CD45RA+CD62L+ ‘naïve’, CD45RA–CD62L+ central memory (CM), CD45RA–CD62L– effector memory (EM) and CD45RA+CD62L– terminally differentiated effector memory cells (TEMRA) and found that the proportion of SARS-CoV-2-specific CM Th cells was maintained at approximately 50% on average, while the level of SARS-CoV-2-specific EM Th cells was about 40% within the total SARS-CoV-2-specific Th cell subset. We also compared the frequencies of virus-specific cells of different differentiation status between the samples stimulated with live SARS-CoV-2 and H1N1 viruses (Figure 1). We found that ‘naïve’ cells were increased in H1N1-stimulated samples (Figure 1A), while the level of EM Th cells was higher in SARS-CoV-2-stimulated samples (Figure 1D).

**Figure 2:**
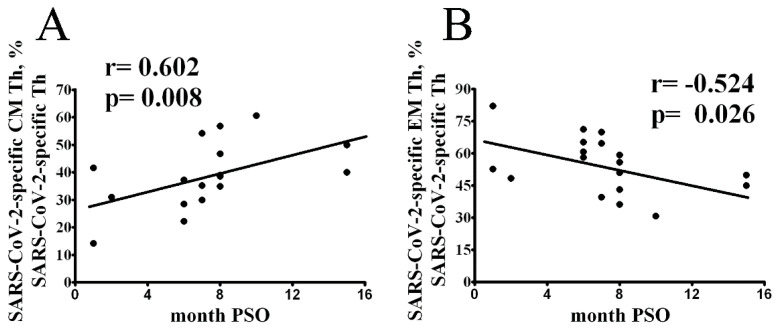
Longitudinal analysis of SARS-CoV-2-specific Th cell frequency and differentiation status. Scatter plots showing the relationship between the frequency of SARS-CoV-2 CM (**A**) and EM (**B**) Th cells within total SARS-CoV-2-specific Th cells. A statistical analysis was performed using the Spearman’s rank-order correlation (*n* = 19).

We further investigated the relationship between the time of post-symptom onset and the proportion of the SARS-CoV-2-specific Th cell subset of differentiation status. Correlative analyses demonstrated that the frequency of SARS-CoV-2-specific CM and EM CD4+ T cell was strongly associated with the time post-symptom onset (Figure 2A,B, respectively).

**Figure 3:**
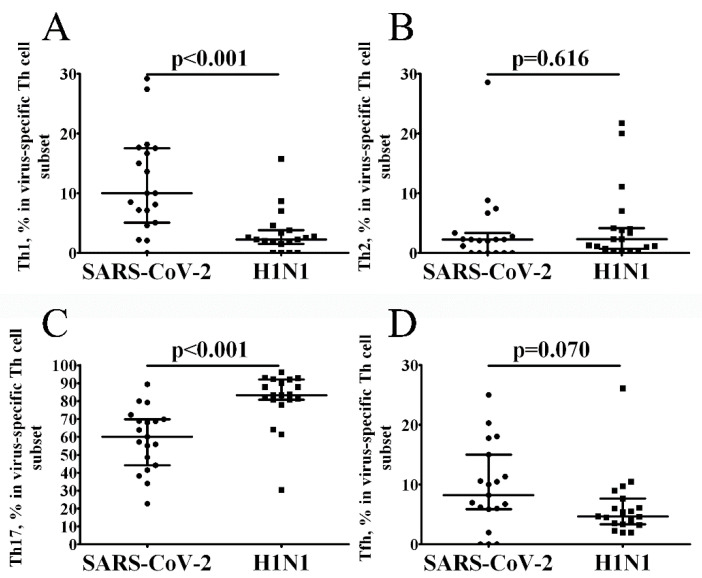
The functional polarization of circulating SARS-CoV-2- and H1N1-specific CD4+ T cells. Scatter plots A-D showing the percentages of Th1 (CXCR5–CCR6–CXCR3+CCR4–) (**A**), Th2 (CXCR5–CCR6–CXCR3–CCR4+) (**B**), Th17 (CXCR5–CCR6+) (**C**) and follicular Th (CXCR5+) (**D**) cells within the total virus-specific Th cell population, respectively. Black circles—SARS-CoV-2-specific CD4+ T cells (*n* = 19); black squares—H1N1-specific CD4+ T cells. Each dot represents individual subjects, and horizontal bars represent the group medians and quartile ranges (Med (Q25; Q75). A statistical analysis was performed with a Mann-Whitney U test.

We also examined the subsets of differentiated CD4+ T cells including Th1, Th2, Th17 and follicular Th (Tfh) cells. Subsets were defined by multicolor flow cytometry as CXCR5–CCR6–CXCR3+CCR4– (Th1), CXCR5–CCR6–CXCR3–CCR4+ (Th2), CXCR5–CCR6+ (Th17) and CXCR5+ (Tfh). No significant differences were detected among groups for Th2 and follicular Th cells (Figure 3B,D, respectively, and Tables S2–S4). We found that SARS-CoV-2-specific CD4+ T cells predominantly expressed Th17 phenotype, but the level of Th17 cells in this group was lower than in H1N1-specific CD4+ T cells (Figure 3C). Furthermore, SARS-CoV-2-specific CD4+ T cells were skewed toward a Th1 cells if compared H1N1-specific CD4+ T cells (Figure 3A). Next, we looked at the relationships between the aforementioned SARS-CoV-2-specific CD4+ T cell subsets and the time of post-symptom onset. We found that only the level of Tfh cells positively correlated with the time PSO (r = 0.449, *p* = 0.041). However, there were no significant correlations between the frequencies of Th1, Th2 and Th17 and the time post-symptom onset (r = 0.054, *p* = 0.816; r = 0.099, *p* = 0.667 and r = 0.270, *p* = 0.236, respectively).

**Figure 4:**
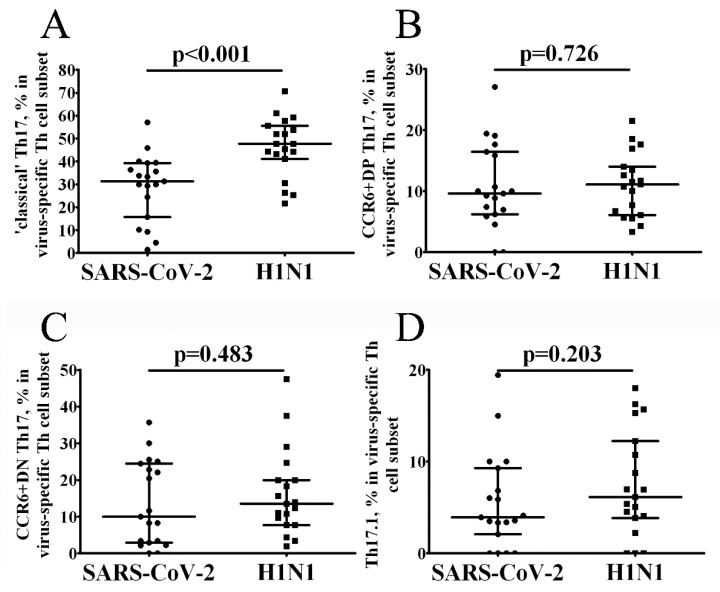
Frequency of circulating SARS-CoV-2- and H1N1-specific Th17 cell. Scatter plots A-D showing the percentages of CCR4+CXCR3– ‘classical’ Th17 (**A**), CCR4+CXCR3+CCR6+ DP Th17 (**B**), CCR4–CXCR3–CCR6+ DN Th17 (**C**) and CCR4–CXCR3+ Th17.1 (**D**) within the total virus-specific Th cell population, respectively. Black circles—SARS-CoV-2-specific CD4+ T cells (*n* = 19); black squares—H1N1-specific CD4+ T cells. Each dot represents individual subjects, and horizontal bars represent the group medians and quartile ranges (Med (Q25; Q75). Statistical analysis was performed with the Mann-Whitney U test.

Thus, SARS-CoV-2-specific CD4+ T cells predominantly expressed the Th17 phenotype. We distinguished four distinct Th17 subsets within the total pool of CCR6-expressing Th17 cells to better define the features of SARS-CoV-2-specific Th17 cells. Previously, based on the differential expression of CCR4 and CXCR3, Wacleche et al. identified CCR4+CXCR3– ‘classical’ Th17, CCR4–CXCR3+ ‘non-classical or Th17.1, CCR4–CXCR3– double negative or CCR6 + DN Th17 and co-expressing CXCR3 and CCR4 double positive or CCR6+ DP Th17 [22]. We found that the lower level of total Th17 subset within total SARS-CoV-2-specific CD4+ T cells was linked with the low level of ‘classical’ Th17 cells if compared with H1N1-specific Th17 cells (Figure 4, Tables S2–S4).

**Figure 5:**
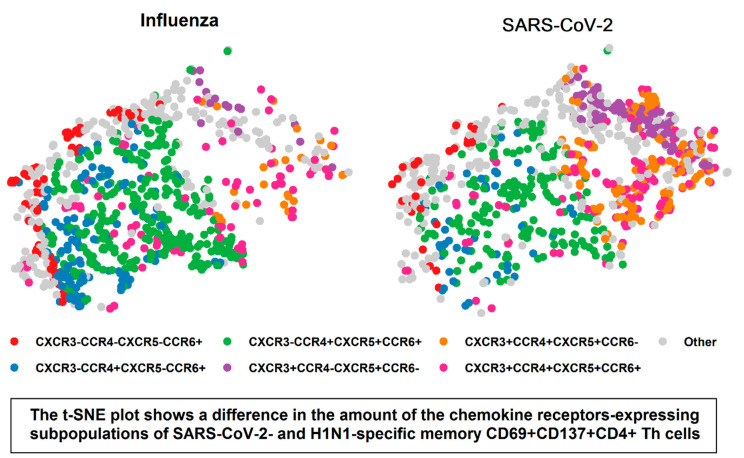
The t-SNE plot of different chemokine receptors-expressing subsets of SARS-CoV-2- and influenza-specific memory CD69+CD137+ CD4+ Th cells. The t-SNE analysis was performed using the cell fluorescence data from H1N1 and SARS-CoV-2-stimulated samples. A random sample (*n* = 800) was selected from each group. t-SNE was performed using R version 4.0.0. The perplexity parameter was set to 40. The iteration number was 1500.

These differences in the amount of the chemokine receptors-expressing subpopulations of SARS-CoV-2- and influenza-specific memory CD69+CD137+ CD4+ Th cells can be visualized using the t-SNE analysis (Figure 5). The visualization shows that in SARS-CoV-2-stimulated samples, the predominant subpopulations of CD69+CD137+ CD4+ Th cells were represented by CXCR3+CCR4–CXCR5+CCR6– and CXCR3+CCR4+CXCR5+CCR6– cells, whereas in influenza-stimulated samples, the majority of memory Th-lymphocytes consisted of CXCR3–CCR4+CXCR5+CCR6+ and CXCR3–CCR4+CXCR5–CCR6+ subsets. Among the above-mentioned phenotypes, CXCR3+CCR4–CXCR5+CCR6–, CXCR3+CCR4+CXCR5+CCR6– and CXCR3–CCR4+CXCR5+CCR6+ correspond to the different subpopulation of circulating Tfh cells, while CXCR3–CCR4+CXCR5–CCR6+ represents subpopulation of Th17 lymphocytes with immunosuppressive activity [23]. The predominance of CXCR3+ cells in SARS-CoV-2-stimulated samples indicates a higher level of Th1-polarisation of circulating Tfh in this group.

## 4. Discussion

Currently, many authors use in vitro stimulation with SARS-CoV-2 peptide pools, followed by quantitation of antigen-specific cells by T cell receptor activation-induced cell-surface molecules, including different types of activation and/or co-stimulation/co-inhibitory receptors, to measure SARS-CoV-2-specific CD4+ T cells [10,18,24]. During our experiment, we also revealed SARS-CoV-2-specific CD4+ T cells by CD69 and CD137 co-expression, but here we used live SARS-CoV-2 virus for the in vitro stimulation of T cells, meaning that the whole viral proteome was used for antigen presentation. It was shown that multiple cell types (including different types of immune cells—CD8+ T cells, monocytes and macrophages) collected from patients with severe COVID-19 contained SARS-CoV-2 viral RNA, indicating that they were infected or they could engulf virus [25]. Moreover, live SARS-CoV-2 effectively infected multiple cell types including macrophages, neutrophils, plasma B cells, and T lymphocytes during in vitro experiments and convalescent sera from COVID-19 patients improved the efficiency of infection [26]. Thus, these data indicated that SARS-CoV-2 was able to penetrate different cells and that a high viral load could cause the induction of apoptosis [27]. However, for effective T cell stimulation with live virus, viral particles need to be internalized, processed and loaded on MHC Class II molecules to make viral antigens available for T cell recognition. Furthermore, a study by Pontelli et al. found that live virus was found mainly in CD14+ monocytes and CD19+ B cells (both subsets were capable for endocytosis and could present internalized antigens on MHC II molecules to Th cells) upon in vitro infection of PBMCs with SARS-CoV-2, whereas infection of CD4+ T lymphocytes occurred less frequently [28], suggesting that the stimulation protocol used in our study is relevant for identification of virus-specific Th subsets in circulation.

Recent studies have shown that SARS-CoV-2-specific CD4+ T cell responses are maintained up to 10–12 months following infection [29,30] or even more, because we were able to identify virus-specific Th cells 15 months PSO in several individuals. Furthermore, detection of SARS-CoV-2-specific stem cell-like memory T cells (CCR7+CD45RA+CD95+) indicate that SARS-CoV-2-specific T-cell memory could be long-lasting [20,31]. We also found that the phenotype of SARS-CoV-2-specific CD4+ T cells could change over time, which was associated with an increased fraction of central memory cells and decreased fraction of effector memory Th cells. However, in the acute phase of the disease the majority of SARS-CoV-2-specific CD4+ T cells in COVID-19 convalescents were identified as central memory T cells [7]. Similarly, longitudinal analysis of Spike-specific CD4+ T cells revealed that effector memory Th cells were gradually replaced by the Th cell with CD45RA–CCR7+ central memory phenotype in convalescent peripheral blood samples [32]. Interestingly, Jung et al. revealed similar kinetics of memory Th cell subsets and noticed that among SARS-CoV-2-specific CD4+ T cells, the proportion of CM cells was rather stable and maintained at approximately 50% during 1–10 months PSO, while the proportion of CCR7–CD45RA– cells increased up to ~35% until two months PSO and then maintained thereafter [20]. Furthermore, the SARS-CoV-specific CD4+ T cells from the convalescent patients tended to be a CD27+CD45RO+ central memory phenotype with a higher frequency of polyfunctional memory cells producing IFNγ, TNFα and IL-2 [11].

It was noticed that during acute COVID-19 SARS-CoV-2-specific CD4+ T cells predominantly produced effector and Th1 cytokines, although Th2 and Th17 cytokines were also detected [7]. The existence of SARS-CoV-2-specific CD4+ Th1 cells was confirmed by demonstrating that Tbet-expressing CD4+ T cells from convalescent individuals (1–2 months PSO) secrete IFNγ in response to in vitro SARS-CoV-2 peptide treatment [33]. When SARS-CoV-2-specific CD4+ T cell subsets were determined after peptide pool stimulation according to the production of intracellular cytokines, the percentages of IFNγ+CD4+ Th1 cells were significantly higher than those of IL-17+CD4+ Th17 cells, while the levels of IL-4+CD4+ Th2 cells were extremely low [29]. Conversely, when an activation-induced molecules assay was used to determine the functional polarization of SARS-CoV-2-specific CD4+ T cells, it was found that S-specific CD4+ T cells were skewed toward a circulating Tfh profile, whereas M- and S-specific CD4+ T cells were skewed toward a Th1 or a Th17.1 profile [34]. These data show that both the method of measurement and in vitro stimulation conditions had a significant impact on the data obtained. Correspondingly, our results showed that in response to live SARS-CoV-2 stimulation, virus-specific Th cells were skewed toward a CXCR5–CCR6+ Th17 phenotype. It should be mentioned that measurements of the viral-specific cellular immune response are traditionally based on the frequency of IFNγ-producing Th1 cells using intracellular staining and flow cytometry and are usually based on in vitro stimulation with a specific antigen for 6 or 18–24 h [35]. However, it is known that in humans there have been several incidental observations of delayed kinetics of IL-4, IL-5, and IL-17 secretion by memory T cells compared with IFNγ secretion [36]. For instance, human cytomegalovirus-induced IFNγ spot numbers peaked at 24 h, IL-4 spot counts showed a delayed kinetics and peaked at 48 h, and started to decline by 72 h, while IL-17 production was minimal at 24 h and peaked only at 72 h post stimulation [36]. Moreover, another study also demonstrated that Th1, Th2 and Th17 cells did not produce cytokines synchronously [37]. It was shown that peptide-triggered PBMC IFNγ production could reach close to maximal levels at 6 h, while it required 24 h for protein antigen that needed to be processed and presented on HLA Class II molecules in order to be available for T cell recognition. Furthermore, Th2 cytokines—IL-4 and IL-13—peaked at 48 h and 72 h, respectively, while IL-17 secretion kinetics by Th17 required 72 h post stimulation to reach the peak [37]. Thus, using enzyme-linked immunospot assay (ELISpot), the production of Th1 signature cytokine IFNγ, Th2 signature cytokine IL-4, and Th17 signature cytokine IL-17 was found to have completely different secretion kinetics. Furthermore, these data may clarify the difference between the results of intracellular flow cytometry, pointing to the main part of IFNγ-secreting memory cells in SARS-CoV-2-specific immune response, and the results of an activation induced cell marker assay, provided by Sekine et al. [34] and in the current paper.

Th17 cells and their effector cytokines are involved in host defense against various infections, especially against extracellular bacteria and fungi, and can enhance the protective properties of various epithelial tissues as well as mediate important crosstalk between the immune system and the peripheral tissue [38]. It is considered that inflammation-induced overproduction of SARS-CoV-2-specific Th17 cells during viral infection can downregulate the antiviral response of Th1 and Th2 cells [39]. Primarily, SARS-CoV-2 infection up-regulated the level of IL-6, which promotes the differentiation of ‘naïve’ Th0 cells into Th17 cells [40], leading to increased levels of circulating CCR6+ Th17 in CD4+ T cells, which participated in the cytokine storm, one of the main causes of disease progression [41,42]. Next, patients with acute COVID-19 had elevated numbers of peripheral blood CD4+ T cells simultaneously producing IL-2 and IL-17 than healthy controls in response to in vitro stimulation with anti-CD3/CD28, pointing to effector Th skewing toward the Th17 phenotype [43]. Another study showed that Th17 and Tfh cells were increased in peripheral blood samples from patients with severe COVID-19 [44]. Conversely, during the acute phase of SARS-CoV-2 infection, CD4+ T cells from the lungs of patients with COVID-19 did not increase Th17-associated genes, including RORC, IL17A, and IL17F, and CCR6 expression was significantly reduced in patients with severe COVID-19 [45].

Furthermore, several studies demonstrated increased levels of IL-17 and GM-CSF in peripheral blood and tears of patients with COVID-19, and a higher fraction of Th17 cells in the site of infection—in bronchoalveolar lavage fluid [43,46]. Moreover, high levels of tissue-resident memory-like CD4+ T cells expressing high amounts of the genes encoding the proinflammatory cytokines IL-17A/F and GM-CSF were identified in the lungs of patients with COVID-19 even after clearance of the virus [47]. Similarly, robust Th17 responses were observed in patients with MERS-CoV and SARS-CoV infections [48,49], as well as a strong Th17 response was also observed in acute H1N1 influenza virus infection [50]. These data indicated that Th17 cells played a critical role in COVID-19, so we were able to identify a large number of SARS-CoV-2-specific CD4+ T cells with the Th17 phenotype.

Finally, we found a difference in SARS-CoV-2- and H1N1-specific Th17 cells associated with ‘classical’ Th17 cells capable of producing a huge number of the major effector Th17 cytokines, IL-17 and IL-22 [22]. But as with SARS-CoV-2 infection, a strong Th17 response was also observed in H1N1 influenza virus infection and pro-inflammatory IL-17 functions that can mediate lung damage in severe human influenza infections [51]. Another study reported an elevated Th17 response during the early phase of pandemic H1N1 infection [50]. In contrast, seasonal influenza was associated with a strong cytokine response, including IL-17, while pandemic H1N1 influenza was known to suppress a Th17-mediated immune response [52]. Some studies have observed higher levels of IL-17 in patients who effectively control the infection compared to patients who have severe disease or death; thus, IL-17 has been shown to have a protective effect, indicating the existence of Th17 responses during this disease [53]. Accordingly, Th17 cells and the IL-17 pathway are involved in several forms of viral infections, including H1N1, MERS-CoV, SARS-CoV, and SARS-CoV-2. It is possible that increased Th17 activity, IL-17A signaling, or hyperactivation of Th17 memory cells during pathogen reinfection may play an important role in virus elimination as well as a causal role in unbalanced inflammation and tissue damage.

This study has several limitations. Thus, the sample size in our study was limited due to the availability and willingness of patients to donate large volumes of whole blood for the analyses, as well as the relatively short timeframe of the experimental studies. For this reason, no clear clinical or pathogenetic correlates with the observed CD4+ Th cell subsets could be established, and we were able to note that only Tfh cells positively correlated with the time PSO. Furthermore, our study group consisted of patients with mild and moderate COVID-19 (Table S1), thereby making full-fledged correlation analysis statistically not significant. Another limitation is that our study participants had unknown influenza infection status; however, this study was conducted in a period when SARS-CoV-2 were circulating everywhere, while influenza viruses were virtually absent from circulation [54]. Therefore, it is very likely that the patients did not contract the influenza infection for at least six months prior to COVID-19. On the other hand, it can be assumed that all patients already had either a history of H1N1 influenza (which has been in circulation since 2009) or had been vaccinated with specific influenza vaccines. That is why it was not surprising that they had substantial levels of circulating influenza virus-specific CD4+ memory T cells. Furthermore, in our study on a similar cohort of patients, both COVID-19 positive and COVID-19 naïve groups had similar levels of H1N1 influenza virus-specific CD4+ and CD8+ memory T cells, suggesting the longevity of influenza-induced cellular responses [13]. The ‘activation-induced marker’ assay used in this study to distinguish virus-specific Th subsets also has some limitations, including non-specific surrogate markers and ‘gaiting strategy’. We primarily used CD69 and CD137, with the former antigen being expressed by TCR-specific and non-specifically activated cells, whereas CD137 (or 4-1BB) is a member of the TNF family which mediates the costimulatory function and is expressed predominantly on CD4+ and CD8+ T cells that have been activated via the T cell receptor. Second, we used the strategies described in the previous works on AIM+ antigen-specific T cells for proper ‘gaiting’ [10,18,55]. Here, we used a combination of these two approaches to detect virus-specific CD4+ T cells in order to increase the reliability of the results obtained. Moreover, for SARS-CoV-2-specific CD4+ T cell identification we used only live virus stimulations, without side-by-side comparisons with peptide stimulation, and the different stimulators can potentially affect the levels of SARS-CoV-2-specific CD4+ T cells activation. On the other hand, the vast majority of other research groups used exclusively synthetic viral peptides for in vitro stimulation [7,8,9,10,11,20,29,30,31,32], which could also influence the phenotype of the identified cells and their frequencies. Finally, to classify CD4+ T cell subsets we used ‘gaiting strategy’ (Figures S1–S3) based on chemokine receptors expression patterns, although intracellular cytokine production and intranuclear expression of linage transcription factors could be used to identify such subsets; however, all approaches had some limitations, as previously described.

## 5. Conclusions

According to our data, convalescent patients with mild and moderate COVID-19 formed a broad spectrum of SARS-CoV-2-specific CD4+ T cell subsets, but they predominantly expressed CCR6 and were capable of inducing type 3 immunity on mucosal tissues. Furthermore, increased levels of ‘classical’ CCR4+CXCR3– Th17 within SARS-CoV-2- and H1N1-specific CD4+ T cells capable of secreting IL-17 (recruitment, activation and migration of neutrophils to the site of inflammation) and IL-22 (promotion of epithelial cell homeostasis and antimicrobial defense) also suggest the predominance of type 3 inflammation during immune responses to SARS-CoV-2 and influenza virus. However, mostly type 1 immunity, mediated by antigen-specific Th1 cells and CD8+ T cells, provides an effective response against intracellular microbes, including viruses. Thus, our data indicate a low efficiency of Th1 cell ‘polarization’ during SARS-CoV-2 and influenza virus infections, and may result in a low efficiency of the immune response during reinfection. Finally, our findings support the involvement of Th17 cells and their separate subsets in the pathogenesis of pneumonia caused by SARS-CoV-2 and H1N1, and a better understanding of Th17 mediated antiviral immune responses may lead to the development of new effective therapeutic strategies.

## Data Availability

The data presented in this study are available on request from the corresponding author.

## Supplementary Materials

The following supporting information can be downloaded at: https://www.mdpi.com/article/10.3390/vaccines10091544/s1, Table S1: Demographic characteristics and SARS-CoV-2-specific antibody immunity of study participants; Table S2: “Polarization” of total circulating SARS-CoV-2- and H1N1-specific CD4+ T cells vs. total conventional CD4+ T cells; Table S3: “Polarization” of CD45RA–CD62L+ central memory circulating SARS-CoV-2- and H1N1-specific CD4+ T cells vs. total central memory CD4+ T cells; Table S4: “Polarization” of CD45RA–CD62L– effector memory circulating SARS-CoV-2- and H1N1-specific CD4+ T cells vs. total effector memory CD4+ T cells. Figure S1: Gaiting of main Th cell subsets; Figure S2: Identification of H1N1- and SARS-CoV-2-specific Th cells; Figure S3: An example of the relative distribution of total Th cells, CM and EM Th cells subsets.
