# Supplementary material for: In Vitro Stimulation with Live SARS-CoV-2 Suggests Th17 Dominance In Virus-Specific CD4+ T Cell Response after COVID-19
Source: Vaccines (Basel). 2022 Sep 16;10(9):1544. doi: 10.3390/vaccines10091544 (PMC9502469; doi:10.3390/vaccines10091544)
Supplement: Supplementary file 1 [file vaccines-10-01544-s001.zip › vaccines-1852044-Supplementary.pdf]

Supplementary Materials

Table S1. Demographic characteristics and SARS-CoV-2-specific antibody immunity of study participants

Subject №	Age, years	Gender	Disease severity	Time post symptoms onset	IgG endpoint titer	MN_{50} titer
1	45	F	mild	1 month	1280	42.6
2	64	F	mild	1 month	2560	145.2
3	37	F	mild	1 month	320	121.7
4	37	F	mild	2 months	2560	38.5
5	39	M	moderate	6 months	1280	17.8
6	40	F	mild	6 months	160	30.0
7	42	M	mild	6 months	1280	10.2
8	24	F	mild	6 months	640	12.1
9	35	F	mild	7 months	1280	27.7
10	34	F	moderate	7 months	640	30.6
11	74	M	moderate	7 months	1280	32.0
12	26	F	moderate	8 months	1280	121.1
13	44	M	moderate	8 months	1280	183.4
14	51	F	mild	8 months	640	<10.0
15	41	F	moderate	8 months	1280	50.6
16	44	M	mild	8 months	640	13.7
17	23	F	mild	10 months	320	<10.0
18	28	F	mild	15 months	640	18.8
19	28	M	mild	15 months	320	<10.0

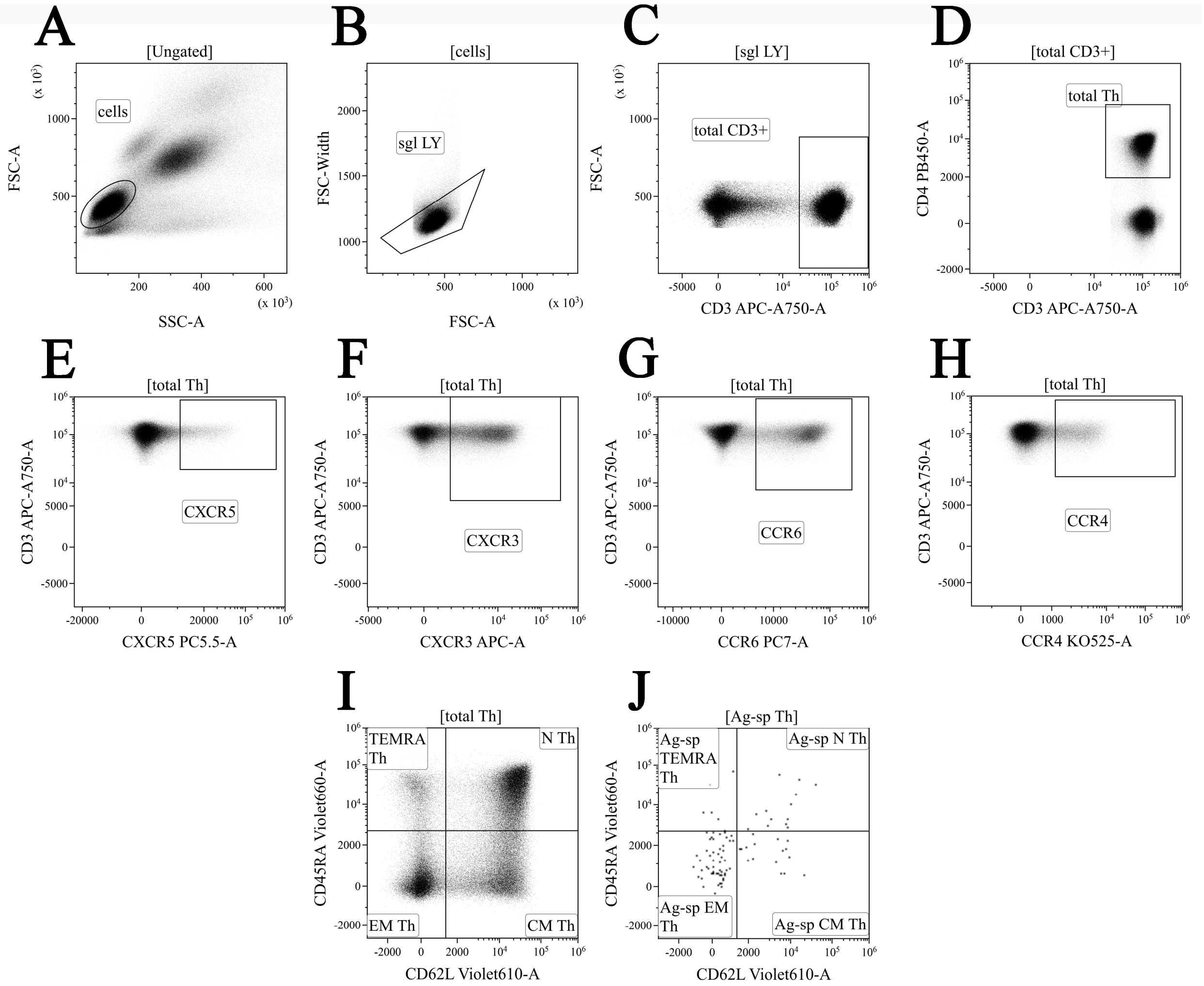

Figure S1. Gaiting of main Th cell subsets. Dot plot A – total lymphocyte subset identification within PBMCs; dot plot B – duplets discrimination; dot plot C – CD3-expressing T cell subsets identification within total lymphocytes; dot plot D – CD3+CD4+ T-helper cell subset identification within total T cells; dot plots E – H – CXCR5, CXCR3, CCR6, and CCR4 expression by Th cells, gates 'CXCR5', 'CXCR3', 'CCR6', and 'CCR4' were used to categorize virus-specific Th cell populations based on whether they have a negative or positive result for a specified Th cell subsets phenotypic. These gates were used as 'branches' for hierarch tree plots shown on Figure S3. Dot plots I and J – CD45RA and CD62L co-expression allowed us to identify four main Th cell maturation subsets: 'naïve' CD45RA+CD62L+ (N Th), central memory CD45RA–CD62L+ (CM Th), effector memory CD45RA–CD62L– (EM Th) and terminally-differentiated CD45RA-positive effector memory CD45RA+CD62L– (TEMRA Th) Th cells. Since the level of virus-specific Th cell subsets was very low in all samples, we used total Th cell subsets for Th cell maturation gaiting, and we used linked gates for dot plots I and J.

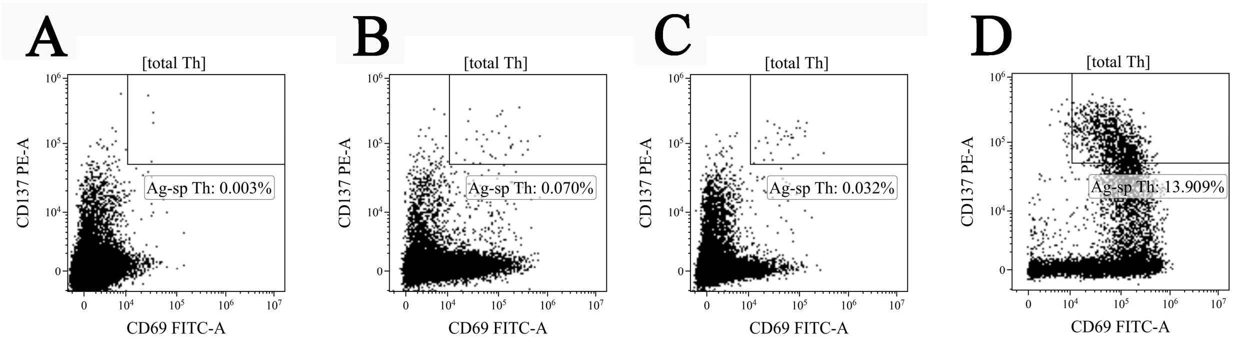

Figure S2. Identification of H1N1- and SARS-CoV-2-specific Th cells. Representative flow cytometry dot plots of SARS-CoV-2-specific CD4+ T cells (CD69+CD137+) after overnight stimulation with H1N1 influenza virus (dot plot B), SARS-CoV-2 (dot plot C) compared to negative unstimulated control (dot plot A) and positive PMA-stimulated control (dot plot D).

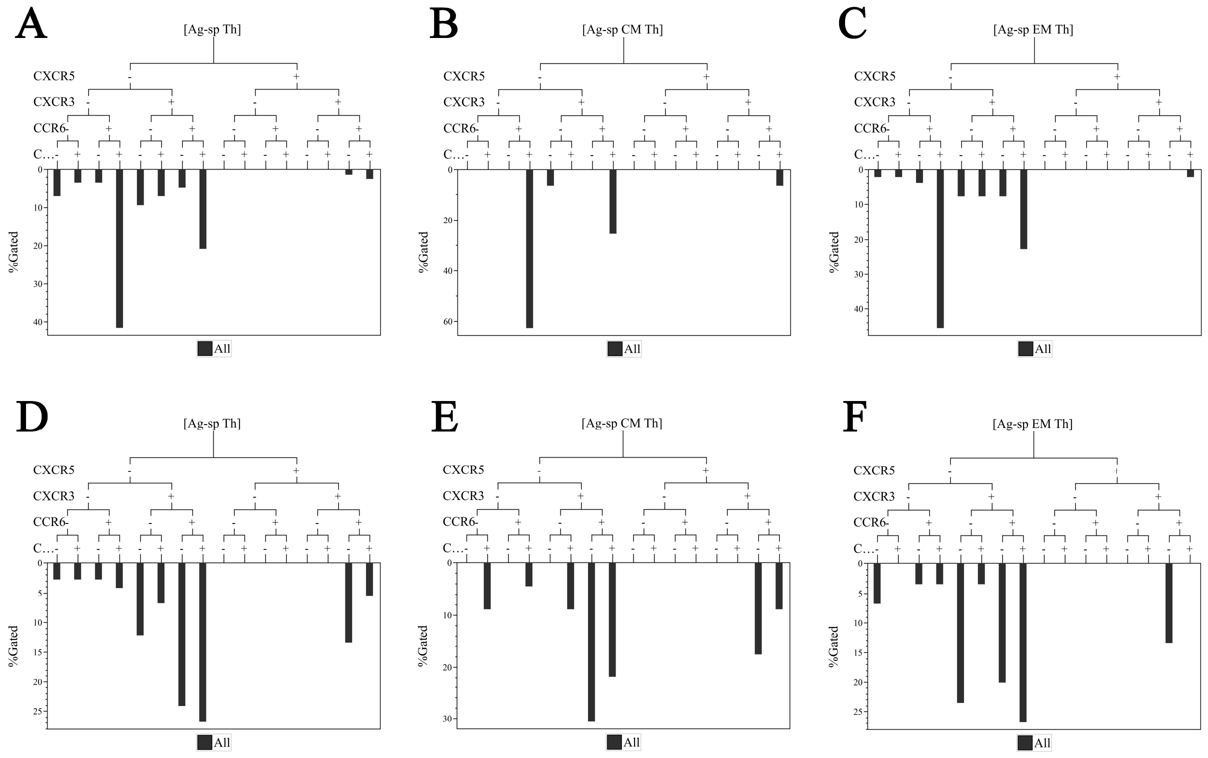

Figure S3. An example of the relative distribution of total Th cells, CM and EM Th cells subsets according to their expression of CXCR5, CXCR3, CCR6, and CCR4 in response to H1N1 (plots A – C) and SARS-CoV-2 (plots D – F) overnight stimulation. Plots A and D – total virus-specific Th cell subset; plots B and E – central memory CD45RA–CD62L+ Th cells, and plots C and F – effector memory CD45RA–CD62L– Th cells. The frequency histogram below the trees indicates the relative proportion of cells with different patterns of CXCR5, CXCR3, CCR6, and CCR4 and is based on their expression in Th cell subsets (as it was shown on dot plots E – H, Figure S1).

Table S2. "Polarization" of total circulating SARS-CoV-2- and H1N1-specific CD4+ T cells vs. total conventional CD4+ T cells. The data are represented group medians and quartile ranges (Med (Q25; Q75), n = 19 for all types of CD4+ T cells

CD4+ T cell subsets	SARS-CoV-2- specific CD4+ T cells	H1N1-specific CD4+ T cells	Total CD4+ T cells	Mann-Whitney U test
**Th1 cells**	10.00 (5.08; 17.53)	2.27 (1.54; 3.85)	18.27 (14.34; 23.77)	$p_1 < 0.001$<br>$p_2 = 0.008$<br>$p_3 < 0.001$
**Th2 cells**	2.25 (0.00; 3.39)	2.31 (0.74; 4.17)	4.28 (3.30; 7.07)	$p_1 = 0.616$<br>$p_2 = 0.003$<br>$p_3 = 0.030$
**Total Th17 cells**	60.00 (44.07; 69.76)	83.33 (80.76; 92.05)	28.20 (22.73; 33.29)	$p_1 < 0.001$<br>$p_2 < 0.001$<br>$p_3 < 0.001$
**DN Th17**	10.00 (2.94; 24.44)	13.51 (7.69; 20.00)	3.57 (3.16; 5.96)	$p_1 = 0.483$<br>$p_2 = 0.077$<br>$p_3 = 0.001$
**'classical' Th17**	31.40 (15.73; 39.29)	47.69 (41.18; 55.56)	7.11 (5.43; 9.68)	$p_1 = 0.001$<br>$p_2 < 0.001$<br>$p_3 < 0.001$
**Th17.1**	3.92 (2.08; 9.30)	6.15 (3.85; 12.26)	10.95 (6.33; 17.34)	$p_1 = 0.203$<br>$p_2 = 0.002$<br>$p_3 = 0.070$
**CCR6+DP Th17**	9.64 (6.25; 16.49)	11.11 (6.12; 14.04)	3.56 (2.12; 4.54)	$p_1 = 0.726$<br>$p_2 < 0.001$<br>$p_3 < 0.001$
**Tfh cells**	8.24 (5.88; 15.00)	4.66 (3.38; 7.70)	17.69 (13.63; 23.53)	$p_1 = 0.070$<br>$p_2 < 0.001$<br>$p_3 < 0.001$

Note: in Supplementary Tables S2 – S4, Th1 cells were CXCR5–CCR6-CXCR3+CCR4–, Th2 were CXCR5–CCR6–CXCR3–CCR4+, total Th17 were CXCR5–CCR6+, 'classical' Th17 cells were CXCR5–CCR6+CCR4+CXCR3-, DP Th17 cells were CXCR5–CCR6+CCR4+CXCR3+, CCR6+DN Th17 cells were CXCR5–CCR6+CCR4–CXCR3–, Th17.1 cells were CXCR5–CCR6+CCR4–CXCR3+ and follicular Th (Tfh) cells were CXCR5+; p_1 – statistical differences between acute SARS-CoV-2- specific CD4+ T cells and H1N1-specific CD4+ T cells assessed by using nonparametric Mann–Whitney U test; p_2 – statistical differences between SARS-CoV-2- specific CD4+ T cells and total conventional CD4+ T cells assessed by using nonparametric Mann–Whitney U tests; p_3 – statistical differences between H1N1-specific CD4+ T cells and total conventional CD4+ T cells assessed by using nonparametric Mann-Whitney U tests.

Table S3. “Polarization” of CD45RA–CD62L+ central memory circulating SARS-CoV-2- and H1N1-specific CD4+ T cells vs. total central memory CD4+ T cells. The data are represented group medians and quartile ranges (Med (Q25; Q75), n = 19 for all types of CD4+ T cells.

CD4+ T cell subsets	SARS-CoV-2- specific CM CD4+ T cells	H1N1-specific CM CD4+ T cells	CM CD4+ T cells	Mann-Whitney U test
**Th1 cells**	8,70 (0,00; 12,50)	0,00 (0,00; 2,90)	18,89 (14,58; 21,37)	$p_1 = 0{,}019$<br>$p_2 < 0{,}001$<br>$p_3 < 0{,}001$
**Th2 cells**	3,85 (0,00; 5,88)	2,78 (0,00; 8,33)	9,18 (7,79; 19,13)	$p_1 = 0{,}702$<br>$p_2 < 0{,}001$<br>$p_3 = 0{,}001$
**Total Th17 cells**	52,38 (33,33; 68,75)	84,06 (74,99; 88,89)	43,11 (37,64; 46,32)	$p_1 = 0{,}001$<br>$p_2 = 0{,}082$<br>$p_3 < 0{,}001$
**DN Th17**	5,41 (0,00; 16,22)	7,69 (4,35; 16,42)	5,96 (4,58; 8,50)	$p_1 = 0{,}577$<br>$p_2 = 0{,}758$<br>$p_3 = 0{,}243$
**‘classical’ Th17**	33,33 (10,00; 41,18)	53,85 (42,03; 57,89)	13,77 (11,70; 16,08)	$p_1 < 0{,}001$<br>$p_2 = 0{,}012$<br>$p_3 < 0{,}001$
**Th17.1**	0,00 (0,00; 3,33)	4,76 (2,78; 9,09)	14,43 (9,24; 18,86)	$p_1 = 0{,}008$<br>$p_2 < 0{,}001$<br>$p_3 < 0{,}001$
**CCR6+DP Th17**	8,77 (0,00; 15,79)	12,50 (8,33; 19,35)	6,96 (4,63; 7,86)	$p_1 = 0{,}137$<br>$p_2 = 0{,}388$<br>$p_3 = 0{,}002$
**Tfh cells**	12,27 (5,40; 21,75)	6,66 (3,23; 10,15)	13,01 (8,96; 18,92)	$p_1 = 0{,}108$<br>$p_2 = 0{,}630$<br>$p_3 = 0{,}003$

Table S4. "Polarization" of CD45RA–CD62L– effector memory circulating SARS-CoV-2- and H1N1-specific CD4+ T cells vs. total effector memory CD4+ T cells. The data are represented group medians and quartile ranges (Med (Q25; Q75), n = 19 for all types of CD4+ T cells.

CD4+ T cell subsets	SARS-CoV-2- specific EM CD4+ T cells	H1N1-specific EM CD4+ T cells	EM CD4+ T cells	Mann-Whitney U test
**Th1 cells**	10,34 (4,35; 19,15)	2,75 (0,00; 5,45)	23,49 (17,76; 37,27)	p_1 = 0,003<br>p_2 < 0,001<br>p_3 < 0,001
**Th2 cells**	0,00 (0,00; 2,13)	0,00 (0,00; 2,56)	2,21 (1,32; 4,17)	p_1 = 0,743<br>p_2 = 0,002<br>p_3 = 0,008
**Total Th17 cells**	68,00 (48,94; 80,00)	85,71 (81,82; 93,59)	47,46 (39,57; 59,09)	p_1 = 0,001<br>p_2 = 0,006<br>p_3 < 0,001
**DN Th17**	14,89 (3,03; 29,55)	23,64 (13,74; 28,85)	6,51 (4,47; 8,49)	p_1 = 0,286<br>p_2 = 0,179<br>p_3 = 0,001
**'classical' Th17**	30,43 (12,77; 36,84)	41,67 (27,78; 52,75)	9,85 (6,66; 14,71)	p_1 = 0,020<br>p_2 = 0,001<br>p_3 < 0,001
**Th17.1**	6,38 (3,92; 10,53)	7,69 (0,00; 13,74)	26,01 (14,99; 37,05)	p_1 = 0,758<br>p_2 < 0,001<br>p_3 < 0,001
**CCR6+DP Th17**	11,36 (0,00; 21,28)	9,09 (4,76; 12,96)	5,28 (3,85; 7,53)	p_1 = 0,758<br>p_2 = 0,260<br>p_3 = 0,020
**Tfh cells**	3,92 (0,00; 8,00)	2,38 (0,00; 5,46)	5,39 (2,98; 7,14)	p_1 = 0,859<br>p_2 = 0,285<br>p_3 = 0,068
